# Extracellular CIRP Promotes GPX4-Mediated Ferroptosis in Sepsis

**DOI:** 10.3389/fimmu.2022.903859

**Published:** 2022-06-29

**Authors:** Junji Shimizu, Atsushi Murao, Colleen Nofi, Ping Wang, Monowar Aziz

**Affiliations:** ^1^ Center for Immunology and Inflammation, The Feinstein Institutes for Medical Research, Manhasset, NY, United States; ^2^ Department of Surgery, Zucker School of Medicine at Hofstra/Northwell, Manhasset, NY, United States; ^3^ Department of Molecular Medicine, Zucker School of Medicine at Hofstra/Northwell, Manhasset, NY, United States

**Keywords:** eCIRP, ferroptosis, lung, sepsis, acute lung injury, macrophage, GPX4

## Abstract

Sepsis is characterized by life-threatening organ dysfunction caused by a dysregulated host response to infection. Extracellular cold-inducible RNA-binding protein (eCIRP) is a damage-associated molecular pattern (DAMP) that promotes inflammation and induces cell death *via* apoptosis, NETosis, and/or pyroptosis. Ferroptosis is a form of regulated cell death characterized by the accumulation of lipid peroxide on cellular membranes. We hypothesize that eCIRP induces ferroptosis in macrophages and lung tissue during sepsis. RAW 264.7 cells stimulated with recombinant murine (rm) CIRP significantly decreased the expression of glutathione peroxidase 4 (GPX4), a negative regulator of ferroptosis, and increased lipid reactive oxygen species (ROS) in a TLR4 dependent manner. In TLR4^-/-^ peritoneal macrophages, depression of GPX4 expression and increase in lipid ROS levels were attenuated after rmCIRP-treatment compared to WT macrophages. rmCIRP also induced cell death in RAW 264.7 cells which was corrected by the ferroptosis inhibitor, ferrostatin-1 (Fer-1). Intraperitoneal injection of rmCIRP decreased GPX4 expression and increased lipid ROS in lung tissue, whereas the increase of lipid ROS was reduced by Fer-1 treatment. GPX4 expression was significantly decreased, while malondialdehyde (MDA), iron levels, and injury scores were significantly increased in lungs of WT mice after cecal ligation and puncture (CLP)-induced sepsis compared to CIRP^-/-^ mice. Treatment with C23, a specific eCIRP inhibitor, in CLP mice alleviated the decrease in GPX4 and increase in MDA levels of lung tissue. These findings suggest that eCIRP induces ferroptosis in septic lungs by decreasing GPX4 and increasing lipid ROS. Therefore, regulation of ferroptosis by targeting eCIRP may provide a new therapeutic approach in sepsis and other inflammatory diseases.

## Introduction

Sepsis is the leading cause of death among critically ill patients and a major global medical problem (1). Sepsis affects approximately 19.4 million people, and the number of deaths is approximately 5.3 million annually worldwide (1). The costs associated with managing septic patients in U.S. hospitals are among the highest of all hospital admissions (2). The lungs are particularly susceptible to injury in response to systemic inflammation during sepsis. Sepsis morbidity and mortality are dramatically elevated by acute lung injury (ALI) and acute respiratory distress syndrome (ARDS) (3).

The immune response to sepsis is multifactorial and is often described as consisting of two components. The first component is the hyperinflammatory state, a systemic inflammatory response to infection (4). Damage-associated molecular patterns (DAMPs) interact with pattern recognition receptors expressed on the surface of immunoreactive cells, leading to the release of pro-inflammatory cytokines and chemokines and the mobilization of immune cells to the site of inflammation, resulting in exacerbation of inflammation and organ dysfunction (4). The second component is a state of immunosuppression, a vulnerable phase for secondary infections (5). Regardless of immune and non-immune cells, various forms of cell death play critical roles in both the hyperinflammatory and immunosuppressive states during sepsis (6). Increased and prolonged inflammation during sepsis contributes to both programmed and unprogrammed forms of cell death, which not only cause organ damage but also leads to prolonged states of immunosuppression susceptible to nosocomial infection (7).

Ferroptosis is a form of regulated cell death and is distinct from other types of programmed cell death including, apoptosis, necroptosis, and autophagy (8). Ferroptosis, initially discovered as iron-dependent cell death, is best characterized by the accumulation of reactive oxygen species (ROS) (9). A variety of diseases have been associated with ferroptosis, such as cancer, intracerebral hemorrhage, traumatic brain injury, stroke, ischemia-reperfusion injury, acute kidney injury (AKI) and ALI (10–14). Recently, ferroptosis has also been reported to be involved in sepsis (15, 16). A major player in the mechanism of ferroptosis is glutathione peroxidase 4 (GPX4), which reduces lipid ROS to protect cells from ferroptosis. GPX4 is a lipid repair enzyme catalyzing glutathione (GSH) that oxidizes glutathione disulfide (GSSG), removes lipid peroxides, and protects the cell membrane against peroxidation of polyunsaturated fatty acids. Hence, GPX4 is the essential negative regulator of oxidative damage to inhibit ferroptosis, and its downregulation is the most common, if not only, way to identify ferroptosis (9, 15).

Cold-inducible RNA-binding protein (CIRP) is an 18-kDa RNA chaperone protein mainly found in the nucleus at steady state (17). When cells are under stress conditions, CIRP is released into the extracellular space. Extracellular CIRP (eCIRP) acts as a DAMP to cause inflammation and organ injury in a number of disorders such as sepsis, hemorrhage and ischemia-reperfusion injury (18). Elevated plasma levels of eCIRP correlate with poor outcomes in patients suffering from sepsis (18). eCIRP activates macrophages, neutrophils, and lymphocytes by binding to Toll-like receptor 4 (TLR4) and triggering receptor expressed on myeloid cells-1 (TREM-1) to induce inflammatory responses such as cytokine production, neutrophil extracellular traps (NETs) formation, and Th1 cell differentiation, respectively (4, 19). We have previously shown that eCIRP induces multiple organ dysfunctions including ALI in sepsis (18, 20).

DAMPs, including eCIRP, are closely linked to cell death. DAMPs have been shown to induce different forms of cell death in various cell types (21, 22). Cell death induced by DAMPs result in the release of more DAMPs from dying cells, propagating a vicious cycle (23). A recent study has shown that high-mobility group box-1 (HMGB1), one of the most widely-studied DAMPs, induces ferroptosis *via* the TLR4/NF-κB axis (24), indicating that ferroptosis can be induced directly by DAMPs. eCIRP has been shown to induce different kinds of cell death, such as endothelial cell apoptosis and NETosis in sepsis (25, 26). However, the role of eCIRP in ferroptosis during sepsis has not yet been elucidated. Therefore, we aimed to identify the direct impact of eCIRP on ferroptosis in sepsis. Our results reveal that eCIRP induces ferroptosis (as determined by the downregulation of GPX4 and accumulation of ROS in macrophages), and that eCIRP plays an important role in ferroptosis in the lungs of septic mice. This study provides new insight into the involvement of eCIRP in ferroptosis which may lead to novel therapeutic approaches in treating sepsis.

## Material and Methods

### Mice

Male 8–12-week-old wild-type (WT) C57BL/6 mice were purchased from Charles River (Charles River, Wilmington, MA). C57BL/6 CIRP^−/−^ mice originally obtained from Jun Fujita (Kyoto University, Kyoto, Japan) and C57BL/6 TLR4^−/−^ mice obtained as a gift from K. J. Tracey (The Feinstein Institutes for Medical Research, Manhasset, NY) were bred and maintained in our facility. Mice were housed in a temperature-controlled room on 12 h light cycles and provided standard laboratory chow and water. All experiments were performed following the guidelines for using experimental animals by the National Institutes of Health and were approved by the Institutional Animal Care and Use Committees (IACUC) of The Feinstein Institutes for Medical Research.

### Mouse Model of Sepsis

Polymicrobial sepsis was induced in mice by cecal ligation and puncture (CLP) (27). Mice were anesthetized by 2% isoflurane inhalation. A 1.5 cm midline abdominal incision was made, and the cecum was exposed and ligated 1 cm proximal to the end with a 4-0 silk suture. The ligated distal part of the cecum was punctured twice with a 22-gauge needle and a small amount of feculent material was extruded from the punctures. The cecum and feculent material were returned to the abdomen, and the abdominal wound was closed in two layers. Sham mice underwent only abdominal incisions without ligation and puncture of the cecum. Following surgery, animals received a subcutaneous injection of 0.5 mL of normal saline and were returned to their cages with normal access to food and drinking water. After 20 h CLP, mice were euthanized with CO_2_ asphyxiation and lungs were collected.

### Administration of rmCIRP and Fer-1 in Mice

Mice were injected intraperitoneally (i.p.) with 5 mg/kg recombinant murine CIRP (rmCIRP) and simultaneously with 2 mg/kg ferrostatin-1 (Fer-1, Catalogue No.:SML0583; Sigma-Aldrich, Saint Louis, MO), then returned to their cages. 4 h after i.p. injection, mice were euthanized with CO_2_ asphyxiation, and lungs were harvested.

### *In Vivo* Administration of C23

C23 (GRGFSRGGGDRGYGG) was synthesized by GenScript USA Inc (Piscataway, NJ). After CLP was completed and animals were closed, the treatment group received an intraperitoneal injection of C23 (8 mg/kg), and the vehicle group received an equivalent volume of normal saline.

### Evaluation of Malondialdehyde 

The MDA concentration of lung tissue lysates was assessed using the MDA assay kit (Catalogue No.: MAK085; Sigma-Aldrich) according to the manufacturer’s instructions. The lysates were mixed with thiobarbituric acid, heated at 95°C for 60 min, and cooled to room temperature for 10 min. The lysates were collected, and the absorbance was measured at 532 nm by a microplate spectrophotometer reader (Agilent, Santa Clara, CA).

### Assessment of Iron in the Lungs

Iron levels of lung tissue lysates were assessed by the iron assay kit (Catalogue No.: MAK025, Sigma-Aldrich) according to the manufacturer’s instructions. The total (ferrous and ferric) iron levels were measured at the absorbance of 593 nm using a microplate spectrophotometer reader (Agilent, Santa Clara, CA).

### Cell Culture

Mouse macrophage cell line RAW 264.7 cells were obtained from ATCC and cultured in Dulbecco’s modified Eagle medium (DMEM) containing 10% FBS, 2 mM glutamine, and 100 IU/mL penicillin-streptomycin. The cells were cultured at 37°C in 5% CO_2_.

### *In Vitro* Treatment With rmCIRP and Fer-1

RAW 264.7 cells were stimulated with 1 μg/mL of rmCIRP for 1, 4, or 20 h, and were also stimulated with 0.01, 0.1, or 1 μg/mL of rmCIRP for 20 h, and GPX4 protein expression was assessed by Western blotting. RAW 264.7 cells were stimulated with 1 μg/mL of rmCIRP with or without 1 μM Fer-1 treatment for 20 h. Samples were collected and centrifuged for 10 min at 400 g and resuspended in 1 mL of PBS. A total of 0.1 × 10 ^6^ cells were suspended in 1 mL of PBS and stained with 1 μL C11-BODIPY (Catalogue No.: D3861; Thermo Fisher, Waltham, MA) for 20 min or LIVE/DEAD fixable stain kit (Catalogue No.: L34955; Thermo Fisher) for 30 min at 4°C. The acquisition was performed on 10,000 events using a BD FAC Symphony flow cytometer (BD Biosciences, San Jose, CA) and data were analyzed with FlowJo software (Tree Star, Ashland, OR).

### *In Vitro* Study of TLR4 Pathway

For inhibition of TLR4, RAW 264.7 cells were pretreated with anti-mouse TLR4 Ab (Catalogue No.: 117602; Biolegend, San Diego, CA) at a dose of 10 μg/mL or isotype IgG control (Catalogue No.: MAB006; R&D Systems, Minneapolis, MN) at a dose of 10 μg/mL for 30 min, followed by stimulation with 1 μg/mL rmCIRP for 20 h. Peritoneal macrophages isolated from WT and TLR4^-/-^ mice were stimulated with 1 μg/mL rmCIRP for 20 h. Expression of GPX4 was assessed by Western blotting and lipid ROS was assessed by flow cytometry.

### Western Blot Analysis

RAW 264.7 cells and homogenized lung tissue were lysed in RIPA buffer, 10 mM Tris buffer (pH 7.5 containing 0.1% Triton-X 100), 1 mM EDTA, 1 mM EGTA, protease inhibitor tablet (Catalogue No.: A32963; Thermo Fisher), and phosphatase inhibitor tablet (Catalogue No.: A32957; Thermo Fisher). Protein concentration was determined by Bio-Rad DC protein assay kit (Bio-Rad, Hercules, CA). Tissue and cell lysates were electrophoresed on 4–12% NuPAGE Bis-Tris Ge and transferred to nitrocellulose membranes. The membranes were blocked with 0.1% casein in Tris buffer saline for 1 h and incubated overnight at 4°C with primary antibodies against β-actin (Catalogue No.: A5441; Sigma-Aldrich) and GPX4 (Catalogue No.: 52455; Cell Signaling Technology, Danvers, MA). After washing with TBST for 10 min three times, membranes were incubated with infrared dye-labeled secondary antibodies for 1 h. The Odyssey image system (LI-COR, Lincoln, NE) was used to for detection and intensity measurement of the target bands, which were quantified using Image Studio Lite software (LI-COR).

### Statistical Analysis

Comparisons between two groups were performed utilizing two-tailed Student’s t-tests. Comparisons between multiple groups were analyzed using a one-way analysis of variance (ANOVA) and significance was determined by Student Newman-Keuls (SNK) tests. Statistical significance was set at p<0.05. Data analyses were performed using GraphPad Prism graphing and statistical software (GraphPad Software, San Diego, CA).

## Results

### eCIRP Decreases GPX4 Expression in Macrophages

**Figure 1:**
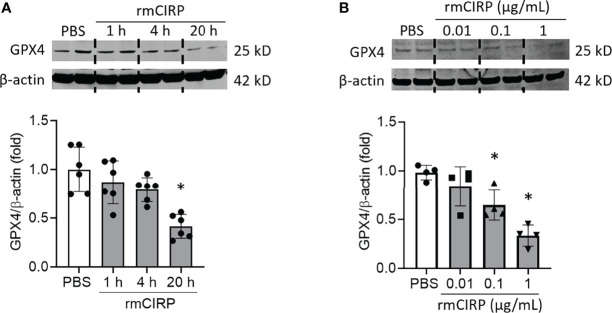
eCIRP downregulates GPX4 expression in macrophages. RAW 264.7 cells were stimulated with **(A)** 1 μg/mL of rmCIRP for 1, 4 and 20 h and **(B)** 0.01, 0.1 and 1 μg/mL of rmCIRP for 20 h. Cells were collected and GPX4 expression was determined by Western blotting. Representative western blot showing GPX4 expression was obtained from a single experiment. The experiments were performed at least 2-3 times, and all the data obtained were analyzed to create the bar diagrams. Data were expressed as mean ± SEM (n = 4-6 sample/group). The groups were compared by one-way ANOVA and SNK method (*p < 0.05 vs. PBS).

GPX4 eliminates lipid ROS and negatively regulates ferroptosis (9). Thus, downregulation of GPX4 is the hallmark of ferroptosis. To examine the effects of eCIRP on GPX4 expression, first, we stimulated RAW 264.7 cells with rmCIRP at different time points and different doses and assessed GPX4 expression. We found that GPX4 expression was significantly decreased in a time-dependent manner. RAW 264.7 cells stimulated with 1 μg/mL rmCIRP for 20 h had decreased GPX4 expression by 58.6% compared to PBS treatment (**Figure 1A**). rmCIRP also dose-dependently induced GPX4 downregulation in RAW 264.7 cells (**Figure 1B**). Thus, eCIRP decreases GPX4 expression in a time- and dose-dependent manner in macrophages.

### eCIRP Induces Ferroptosis in Macrophages

**Figure 2:**
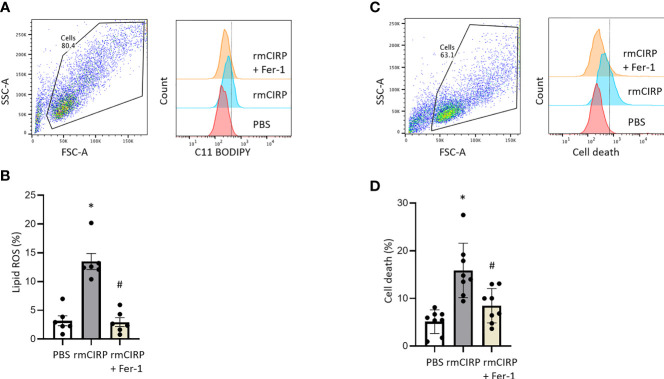
eCIRP increases lipid ROS, and cell death which are attenuated by Fer-1 treatment in macrophages. RAW 264.7 cells were treated with PBS, rmCIRP or rmCIRP + Fer-1 for 20 h. Cells were collected and stained with C11 BODIPY 581/591 and assessed lipid ROS by flow cytometry. **(A)** Dot blots and histograms representing lipid ROS in RAW 264.7 cells are shown. **(B)** A total of 0.1 × 10 ^6^ cells were suspended in 1 mL of PBS and stained with 1 μL C11-BODIPY. Unstained cells were used as a negative control to establish the flow cytometer voltage setting. The FSC-A/FSC-H gating was used to remove doublets. Lipid peroxidation was quantified by the fluorescence intensities in FITC channel. We expressed the data in terms of percentages (%) of cells that were positive for C11-BODIPY staining as represented by the dotted line in the histogram. Quantitative bar diagrammatic presentation of lipid ROS in RAW 264.7 cells in PBS control, rmCIRP stimulation, rmCIRP stimulation with Fer-1 treatment are shown. The experiments were performed 2 times, and all the data obtained were analyzed to create the bar diagrams. Data were expressed as mean ± SEM (n = 6 sample/group). The groups were compared by one-way ANOVA and SNK method (*p < 0.05 vs. PBS; ^#^p < 0.05 vs. rmCIRP). **(C, D)** RAW 264.7 cells were treated with PBS, rmCIRP or rmCIRP + Fer-1 for 20 h. A total of 0.1 × 10 ^6^ cells were suspended in 1 mL of PBS and stained with 1 μL LIVE/DEAD Fixable Violet Dead Cell Stain kit. Unstained cells were used as a negative control to establish the flow cytometer voltage setting. The FSC-A/FSC-H gating was used to remove doublets. Cell death was quantified by the fluorescence intensities in Pacific Blue channel. We expressed the data in terms of percentages (%) of cells that were positive for Live/DEAD Fixable Violet Dead Cell staining as represented by the dotted line in the histogram. **(C)** Dot blots and histograms representing cell death in RAW 264.7 cells are shown. **(D)** Quantitative bar diagrammatic presentation of cell death in PBS control, rmCIRP stimulation, rmCIRP stimulation with Fer-1 treatment are shown. The experiments were performed 2 times, and all the data obtained were analyzed to create the bar diagrams. Data were expressed as mean ± SEM (n = 8 sample/group). The groups were compared by one-way ANOVA and SNK method (*p < 0.05 vs. PBS; ^#^p < 0.05 vs. rmCIRP).

Next, the effect of eCIRP on lipid ROS accumulation, a characteristic feature of ferroptosis, was assessed. After 20 h of rmCIRP stimulation, lipid ROS levels of RAW 264.7 cells were significantly increased by 4.2-fold compared to PBS treatment. The ferroptosis inhibitor, Fer-1, reduced rmCIRP-induced lipid ROS by 78.1%, supporting lipid ROS accumulation was indeed due to ferroptosis (**Figures 2A, B**). We also stimulated RAW 264.7 cells with rmCIRP with or without Fer-1 treatment for 20 h and assessed cell death by flow cytometry. Stimulation of RAW 264.7 cells with rmCIRP significantly increased cell death by 3.1-fold compared to PBS control, while Fer-1 treatment attenuated rmCIRP-induced cell death by 46.5%, indicating ferroptosis is one of the main mechanisms of eCIRP-induced cell death (**Figures 2C, D**). Taken together, this data suggests that eCIRP induces ferroptosis in macrophages.

### eCIRP Induces Ferroptosis in Macrophages *via* TLR4

**Figure 3:**
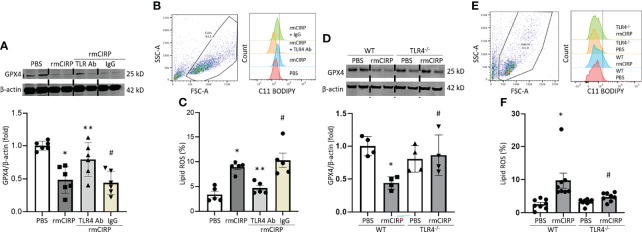
eCIRP causes ferroptosis *via* TLR4 in macrophages. RAW 264.7 cells were treated with PBS, rmCIRP, rmCIRP + TLR4 Ab or rmCIRP + Isotype IgG for 20 h. **(A)** GPX4 expression were determined by Western blotting. **(B)** Dot blots and histograms representing lipid ROS in RAW 264.7 cells are shown. **(C)** Quantitative bar diagrammatic presentation of lipid ROS in PBS control, rmCIRP stimulation, rmCIRP stimulation with TLR4 Ab and rmCIRP stimulation with isotype IgG are shown. Representative western blot showing GPX4 expression was obtained from a single experiment. The experiments were performed at least 2 times, and all the data obtained were analyzed to create the bar diagrams. Data were expressed as mean ± SEM (n = 5-6 sample/group). The groups were compared by one-way ANOVA and SNK method (*p < 0.05 vs. PBS; **p < 0.05 vs. rmCIRP; ^#^p < 0.05 vs. rmCIRP + TLR4 Ab). **(D–F)** Peritoneal macrophages were isolated from both WT and TLR4^-/-^ mice and stimulated with 1 μg/mL rmCIRP for 20 h. **(D)** GPX4 expression was determined by Western blotting. Representative western blot showing GPX4 expression was obtained from a single experiment. **(E)** Dot blots and histograms representing lipid ROS in peritoneal macrophages are shown. **(F)** Quantitative bar diagrammatic presentation of lipid ROS in PBS control, rmCIRP stimulation of WT, and TLR4^-/-^ mice are shown. The experiments were performed at least 2 times, and all the data obtained were analyzed to create the bar diagrams. Data were expressed as mean ± SEM (n = 4-8 sample/group). The groups were compared by one-way ANOVA and SNK method (*p < 0.05 vs. WT PBS; #p < 0.05 vs. WT rmCIRP).

We have previously shown that eCIRP binds to the TLR4-MD2 receptor complex and activates its downstream signaling (18, 25). To investigate the involvement of the TLR4 pathway in eCIRP-induced ferroptosis, we assessed GPX4 expression and lipid ROS of RAW 264.7 cells in rmCIRP-stimulated conditions with or without TLR4 Ab treatment. Pretreatment with TLR4 Ab significantly attenuated the decrease of GPX4 expression following rmCIRP stimulation, while pretreatment with isotype IgG control did not correct the GPX4 downregulation induced by rmCIRP (**Figure 3A**). Pretreatment with TLR4 Ab attenuated the increase of lipid ROS following rmCIRP stimulation, while pretreatment with isotype IgG control did not decrease the rmCIRP-mediated lipid ROS levels (**Figures 3B, C**). We assessed GPX4 expression, and the lipid ROS levels in rmCIRP-treated murine peritoneal macrophages isolated from WT and TLR4^-/-^ macrophages. Our data showed that the treatment of WT mice peritoneal macrophages with rmCIRP significantly decreased the expression of GPX4 compared to PBS-treated conditions. In contrast, the decrease in GPX4 expression in rmCIRP-treated TLR4^-/-^ macrophages was considerably smaller than in WT macrophages (**Figure 3D**). Similarly, the lipid ROS levels were significantly increased in rmCIRP-treated WT macrophages compared to PBS-treated control. However, the lipid ROS levels were significantly decreased in TLR4^-/-^ macrophages compared to WT macrophages after treatment with rmCIRP (**Figures 3E, F**). Taken together, this data suggests that ferroptosis induced by eCIRP is mediated through TLR4 pathway.

### Intraperitoneal Injection of rmCIRP Induces Ferroptosis in Lungs

**Figure 4:**
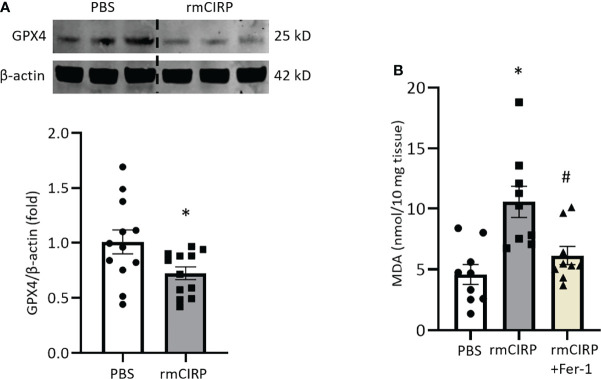
Intraperitoneal injection of rmCIRP induces ferroptosis in lungs. **(A)** Lung tissues were collected after 4 h of intraperitoneal injection of rmCIRP and subjected to Western blotting to assess GPX4 expression. **(B)** Lung tissues were collected after 4 h of intraperitoneal injection of rmCIRP with or without Fer-1 treatment and subjected to MDA assay to assess lipid ROS. Representative western blot showing GPX4 expression was obtained from a single experiment. The experiments were performed at least 3 times, and all the data obtained were analyzed to create the bar diagrams. Data were expressed as mean ± SEM (n = 9-12 mice/group). The groups were compared by Student’s t-test or one-way ANOVA and SNK method (*p < 0.05 vs. PBS; ^#^p < 0.05 vs. rmCIRP).

Next, we investigated whether eCIRP induces ferroptosis *in vivo*. We previously showed that rmCIRP injection was sufficient to induce ALI in mice (28). We injected mice with rmCIRP intraperitoneally and after 4 h of rmCIRP injection, we harvested lung tissue. We found that GPX4 expression in lungs was significantly decreased by 28.2% after rmCIRP injection compared to PBS injection (**Figure 4A**). Lipid ROS, as determined by the MDA levels in the lungs of rmCIRP-injected mice, was significantly increased by 2.3-fold compared to that of PBS-injected mice. Meanwhile, Fer-1 treatment attenuated lipid ROS induced by rmCIRP injection by 41.8% (**Figure 4B**). This data suggests that eCIRP directly induces ferroptosis in the lungs of mice.

### Deficiency of CIRP Protects Mice From Ferroptosis in Lungs During Sepsis

**Figure 5:**
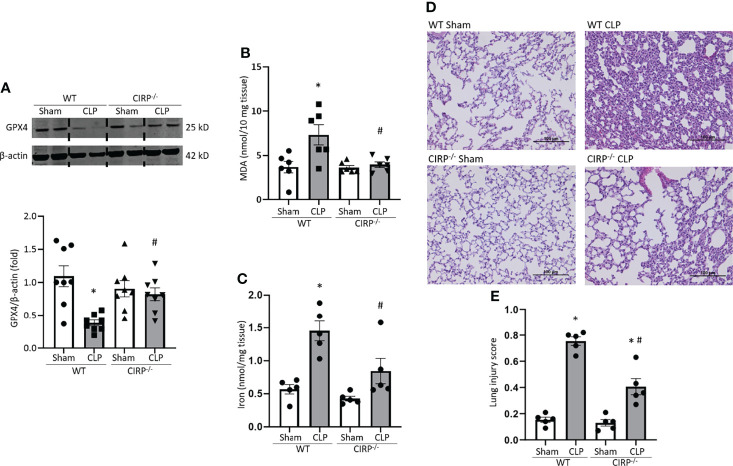
GPX4 expression is reduced, and MDA levels are increased in the lungs after sepsis in WT mice, but not in CIRP^−/−^ mice. After 20 h of CLP or sham procedure, lung tissues were collected from WT and CIRP^−/−^ mice and subjected to **(A)** Western blotting to assess GPX4 expression, **(B)** MDA assay to assess lipid ROS, and **(C)** iron assay. Representative western blot showing GPX4 expression was obtained from a single experiment. The experiments were performed at least 2 times, and all the data obtained were analyzed to create the bar diagrams. Data were expressed as means ± SE (n = 5-8 mice/group). The groups were compared by one-way ANOVA (*p < 0.05 vs. WT sham; ^#^p < 0.05 vs. WT CLP mice). **(D, E)** After 20 h of CLP or sham procedure, lung tissues were collected from WT and CIRP^−/−^ mice. **(D)** Representative images of H&E-stained lung tissue at original magnification ×200. **(E)** Lung injury score calculated at original magnification ×400. n = 5 high-powered fields/group. Data were expressed as means ± SEM. The groups were compared by one-way ANOVA and SNK method (*p < 0.05 vs. WT sham; ^#^p < 0.05 vs. WT CLP mice).

We then sought to determine the role of eCIRP on ferroptosis in septic mice using a CLP model. We induced sepsis in WT and CIRP^-/-^ mice and after 20 h of sepsis, we harvested lung tissue. We found that GPX4 expression in lungs was significantly decreased after CLP by 65% in WT mice. By contrast, CIRP^-/-^ mice barely showed GPX4 downregulation in lungs even though they were subjected to CLP (**Figure 5A**). MDA levels of lungs were significantly increased in WT septic mice by 2.0-fold compared to WT sham mice, whereas MDA levels of CIRP^-/-^ septic mice were significantly lower than that of WT septic mice by 46% (**Figure 5B**). Moreover, iron levels of lung tissue were increased by 2.5-fold after CLP in WT mice, while CIRP^-/-^ mice barely conferred an increase in lung iron levels after CLP (**Figure 5C**). We also determined the overall injury of lung tissue of WT and CIRP^-/-^ septic mice. Our data showed that in CIRP^-/-^ mice, lung injury scores were significantly reduced compared to WT mice (**Figures 5D, E**). This data suggests that eCIRP plays a pivotal role in ferroptosis by decreasing GPX4 and increasing lipid ROS in the lungs during sepsis.

### C23 Treatment Attenuates the Downregulation of GPX4 Expression and Upregulation of ROS in Lungs During Sepsis

**Figure 6:**
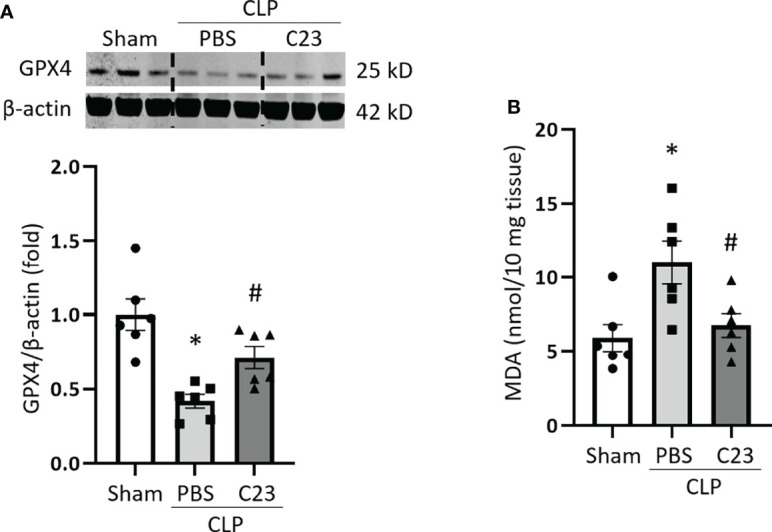
C23 protects mice from ferroptosis in the lungs in sepsis. After 20 h of CLP or sham procedure, lung tissues were collected from sham, vehicle (PBS), and C23 (8 mg/kg) treatment groups. **(A)** GPX4 expression was assessed by Western blotting and **(B)** lipid ROS was assessed by MDA assay. Representative western blot showing GPX4 expression was obtained from a single experiment. The experiments were performed 2 times, and all the data obtained were analyzed to create the bar diagrams. Data were expressed as means ± SE (n = 6 mice/group). The groups were compared by one-way ANOVA (*p < 0.05 vs. sham; ^#^p < 0.05 vs. vehicle).

C23 is an oligopeptide derived from CIRP that binds to the TLR4-MD2 complex and thus acts a competitive inhibitor of eCIRP (18). C23 has already been shown to protect against ALI in septic mice (29) but its effects on ferroptosis have not yet been assessed. Therefore, we evaluated the impact of C23 treatment on the regulation of ferroptosis in sepsis. Mice were injected with 8 mg/kg of C23 or equivalent volume of normal saline at the end of CLP, and after 20 h lung samples were collected. In line with earlier experiments, GPX4 expression was significantly decreased in the lung tissue after CLP. Meanwhile, treatment with C23 significantly restored CLP-induced GPX4 downregulation in lung tissue (**Figure 6A**). MDA levels in the lungs were increased after CLP, while C23 significantly protected mice from elevations in lung MDA after CLP (**Figure 6B**). These results indicate that C23, an inhibitor of eCIRP, alleviates ferroptosis in lungs during sepsis.

## Discussion

**Figure 7:**
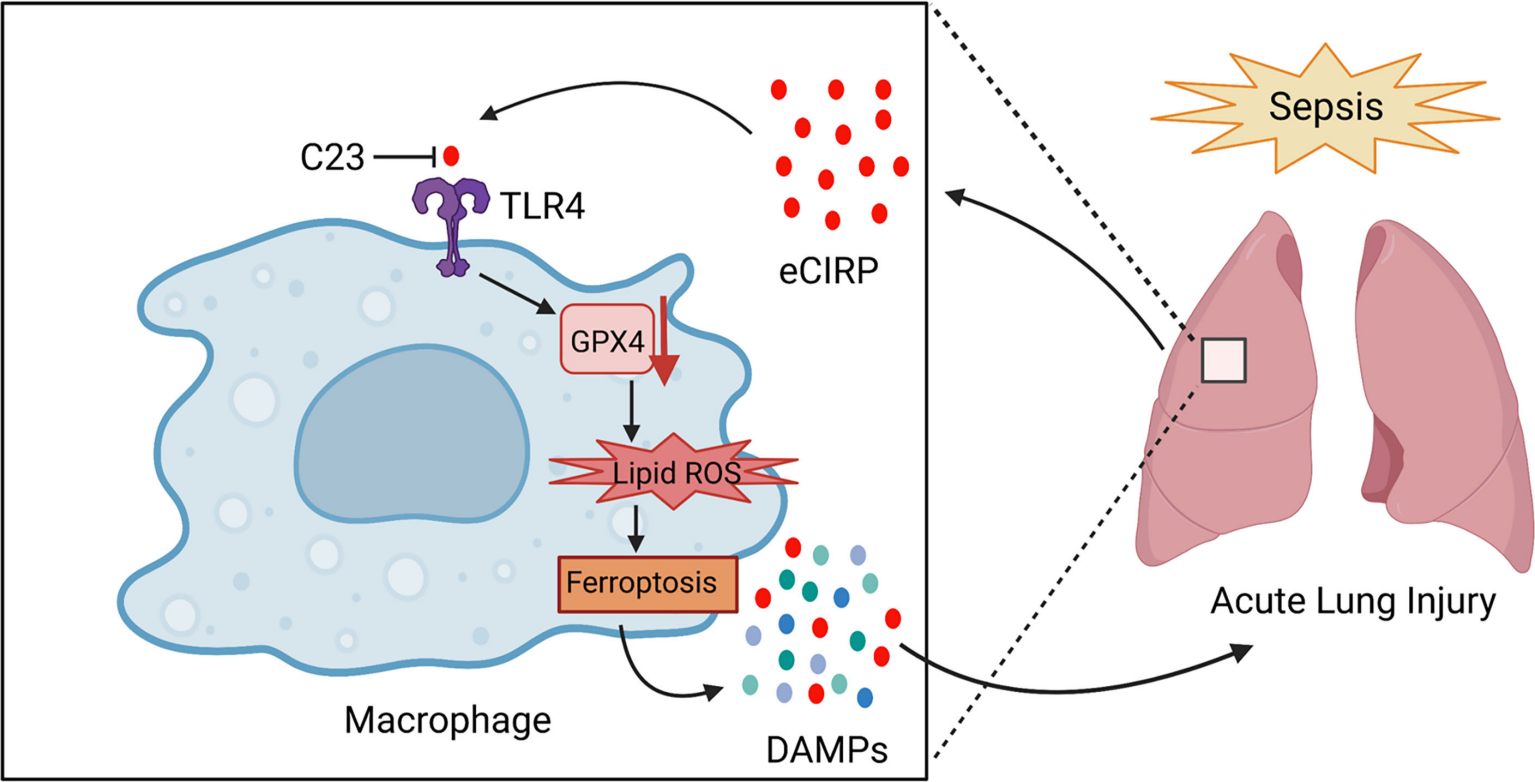
Summary schema.In sepsis, eCIRP is increased in the circulation and organs. eCIRP binds to TLR4 and decreases GPX4 expression, leading to the accumulation of lipid ROS to induce ferroptosis. Ferroptosis causes the release of DAMPs such as eCIRP and contributes to lung injury. C23 acts as an inhibitor of eCIRP through blocking the interaction between eCIRP and TLR4, thus decreasing lung ferroptosis in sepsis. The schema was prepared by BioRender.com.

In the present study, we demonstrate that eCIRP induces ferroptosis *in vitro* and *in vivo* and eCIRP plays an important role in lung ferroptosis during sepsis (**Figure 7**). Ferroptosis is a type of regulated cell death (RCD) caused by the accumulation of lipid ROS in the cell membrane (30). Although any single marker is sufficient to determine ferroptosis, here we adopted several downstream surrogate markers of the GPX4 pathway, i.e., lipid ROS and cell death, to rigorously establish that eCIRP serves as an inducer of ferroptosis in sepsis. GPX4 downregulation is widely used as one of the main factors in identifying ferroptosis (9, 31). GPX4 catalyzes GSH to GSSG, removing lipid ROS and protecting cell membranes from ROS (32). Decreased activity of GPX4 causes accumulation of lipid ROS to induce ferroptosis - thus GPX4 is considered as a central regulator of ferroptosis (33). In this study, we confirmed ferroptosis by combination of GPX4 downregulation, lipid ROS accumulation, and cell death that can be reversed by Fer-1, a ferroptosis inhibitor.

The lungs are susceptible to injury during sepsis, including ALI (3). Excessive accumulation of ROS is thought to be one of the important factors in the pathogenesis of lung injury (34), and cell death also contributes to ALI, suggesting ferroptosis could play an important role. Although apoptosis and secondary necrosis have been considered the major forms of cell death in sepsis-induced ALI (35, 36), emerging evidence has implicated ferroptosis in pathologic sequela of sepsis, including lung injury. Ferroptosis has been associated with lung injury induced by LPS (37) and liver damage and brain dysfunction induced by experimental sepsis, including CLP (15, 38). It has also been reported that GSH, a direct antioxidant and an important substrate for GPX4 to prevent lipid ROS accumulation, is decreased in the alveolar lavage fluid of patients with ALI/ARDS (39). Together with our findings, this supports that eCIRP-induced ferroptosis is an important mechanism in sepsis-induced ALI.

In the current study, we performed time- and dose-dependent effects of rmCIRP on GPX4 expression in RAW 264.7 cells, which revealed that the highest inhibition of GPX4 expression was observed at 20 hours with 1 μg/ml rmCIRP stimulation. Since we observed a profound decrease (>65%) of GPX4 at the highest dose (1 µg/mL) and longest utilized time point (20 h), we did not study eCIRP’s further effects on GPX4’s expression on higher concentrations of rmCIRP or more extended times of incubation. Our previous studies showed that a higher dose of rmCIRP, i.e., 5 µg/mL, significantly increased extracellular trap formation in macrophages and neutrophils (40, 41), suggesting experiments with rmCIRP at higher doses or extended times would likely show greater induction of ferroptosis. However, eCIRP’s effects may vary depending on cell types and signaling pathways of interest, implicating future studies demonstrating its impact on ferroptosis in these extreme conditions. Our previous studies have shown that mice injected with rmCIRP at a dose of 5 mg/kg BW i.v. significantly increased the expression of inflammatory cytokines and chemokines in the lungs, lung histology injury scores, lung neutrophil infiltration, and MPO activity (19, 42). Since we injected mice with the same dose of rmCIRP intraperitoneally (i.p.), we expected that in our rmCIRP-treated mice, lung tissue would similarly exhibit greater injury than in PBS-treated mice. Our previous studies using CIRP^-/-^ mice or WT mice treated with C23 (the antagonist of CIRP) showed significant decreases in the expression of inflammatory cytokines and chemokines in the lungs, lung histology injury scores, neutrophil infiltration, and MPO activity in lungs (18, 29). Following the protocol of our previous studies utilizing CLP-induced sepsis in WT and CIRP^-/-^ mice and treatment with a similar dose of C23, we expect that both CIRP^-/-^ mice and WT mice treated with C23 would exhibit significantly less injury than WT CLP or vehicle-treated septic mice.

Fer-1 is the most commonly used inhibitor of ferroptosis, and its function is primarily through the inhibition of lipid ROS (43). We found that Fer-1 treatment significantly alleviated both lipid ROS accumulation and cell death induced by eCIRP. eCIRP is already known to induce different types of cell death, including apoptosis, NETosis, and pyroptosis (20, 28, 40). Our current study indicates that ferroptosis accounts for a significant proportion of eCIRP-induced cell death by the degree of cell death reduction incurred by Fer-1 treatment in eCIRP-stimulated macrophages. The *in vivo* dose of Ferostatin-1 (Fer-1) was chosen from a previous report (44). Although we did not assess for markers of apoptosis in eCIRP-induced macrophages, eCIRP’s impact on various forms of cell death, including newly identified ferroptosis, implicates its diverse role in cell death. As such, eCIRP-mediated cell death is not strictly limited to a specific modality, but rather is mediated through several pathways. Future studies evaluating the presence of several cell death markers/pathways in ferroptosis cells simultaneously would be helpful to confirm whether, in specific cell types, a particular pathway is activated or overlaps with several mechanisms to induce cell death. This may also uncover the predominance of specific cell death pathways in different cell types during eCIRP stimulation.

We have previously demonstrated that the pro-inflammatory function of eCIRP derives from its activation through the TLR4/MD2 complex on macrophages (25). TLR4-dependent NF-κB signaling has been associated with ferroptosis. Our *in vitro* findings whereby blocking the TLR4 pathway alleviated eCIRP induced GPX4 downregulation and lipid ROS support the role of TLR4 activation in ferroptosis. This was corroborated *in vivo* with the inhibition of ferroptosis utilizing C23. Furthermore, HMBG1 has been shown to induce ferroptosis *via* the TLR4/NF-κB axis (24). The precedent for DAMPs (such as HMGB1) to utilize this pathway of ferroptosis induction further supports the notion that eCIRP utilizes a similar pathway in ferroptosis. Ferroptosis is accompanied by a release of DAMPs and inflammatory cytokines (45, 46). These inflammatory molecules further promote ferroptosis and other forms of cell death, propagating a vicious cycle that causes end-organ damage, including lung injury (47). Elevated plasma levels of eCIRP have been independently correlated with increased disease severity in patients with sepsis (18, 48). Septic mice have also shown to have elevated levels of eCIRP circulating in the blood and found in organs such as the lungs (49). The association between increased ferroptosis and elevated eCIRP levels during sepsis supports ferroptosis as an important mechanism of eCIRP release in sepsis, although further studies are warranted to provide further evidence. Ferroptosis was initially discovered as a mechanism of iron-dependent cell death. However, it is still an open question whether iron is essential for ferroptosis, or other molecules can replace its role (31). Indeed, erastin is often used as an inducer of ferroptosis especially *in vitro* even without iron supplementation to the culture media (50). Thus, in our *in vitro* study we used GPX4 downregulation and lipid ROS accumulation to identify ferroptosis instead of determining whether or not iron dependence is involved. Nonetheless, ferroptosis in cultured macrophages may be iron-dependent, despite the lack of iron supplementation in our culture media since the component, FBS, contains a small amount of iron. Our *in vivo* data showed CIRP^-/-^ mice were protected from iron accumulation in the lung tissues after CLP, indicating eCIRP does play a role in iron-dependent ferroptosis during sepsis.

In animal experiments, we confirmed ferroptosis only in whole lung tissue and did not distinguish between cell types. Nevertheless, the assessment of GPX4 and/or lipid ROS in whole tissue has been used to evaluate organ ferroptosis in previous studies (15, 37). We assessed ferroptosis in the whole lung because of the technical challenge associated with isolating different types of single cells without causing damage. Furthermore, the importance clinically rests on the injury of the organ as a whole and the subsequent dysfunction of that organ. We showed that eCIRP induces ferroptosis through TLR4, suggesting eCIRP has the potential to induce ferroptosis in any cell type expressing TLR4, including immune cells, vascular cells, and endothelial cells (51, 52). Although we did not confirm ferroptosis in cell types other than macrophages, TLR4 expressing cells would likely demonstrate similar effects *in vitro*. Future studies investigating the impact of eCIRP on ferroptosis using alveolar epithelial and endothelial cells, and other cell types, will provide greater insight into the role of eCIRP in inducing ferroptosis globally. Since lungs contain alveolar macrophages, our *in vitro* data with macrophage ferroptosis supports that alveolar macrophage could also be susceptible to ferroptosis in the lungs during sepsis. Our previous reports show that eCIRP induces immune cells, i.e., macrophages, neutrophils, lymphocytes, as well as non-immune cells, like epithelial cells and endothelial cells (25). Given the impact of eCIRP on diverse cell types, we assume that eCIRP can also affect lung epithelial cells to downregulate GPX4 expression to induce ferroptosis. In fact, our recent study revealed a pro-inflammatory effect of eCIRP on primary mouse alveolar type II cells (53), thus supporting the notion that eCIRP could affect other cell types in the lungs to downregulate GPX4 expression.

In our current study, we utilized a model of experimental sepsis following our previous publications utilizing CLP. This model has been shown to induce high levels of pro-inflammatory cytokines, chemokines, lung injury scores, neutrophil infiltration, and LD50 mortality rate (18). Further, it has been demonstrated that these markers of sepsis severity are dramatically improved in CIRP^-/-^ mice. To validate our sepsis model, we utilized lung injury scores in WT and CIRP^-/-^ mice as a representative markers of sepsis severity. Thus, our findings of highest lung injury scores in WT mice (compared to sham) and decrease in lung injury severity in CIRP^-/-^ mice both internally validate our CLP model and demonstrate the impact of eCIRP in sepsis severity.

In our present study, we focused on sepsis-induced ALI and implicate eCIRP-induced ferroptosis as playing a significant role. Both eCIRP and ferroptosis are associated not only with sepsis-induced ALI but also with different organ dysfunctions in various disease conditions (9, 25, 31). Thus, unveiling the mechanism of eCIRP-induced ferroptosis may be implicated in other forms of end-organ damage and pathogenesis of diseases in addition to sepsis-induced ALI. In conclusion, eCIRP induces ferroptosis in lungs in sepsis. Therefore, eCIRP targeted strategies to control ferroptosis may provide a new therapeutic tool in treating sepsis and other acute inflammatory diseases.

## Data Availability Statement

The original contributions presented in the study are included in the article/supplementary material. Further inquiries can be directed to the corresponding authors.

## Ethics Statement

The animal study was reviewed and approved by Institutional Animal Care and Use Committees (IACUC) of the Feinstein Institutes for Medical Research.

## Author Contributions

JS, AM, and MA designed experiments. JS performed experiments. All authors analyzed the data and actively participated in revising the manuscript. JS, AM, and MA wrote the initial manuscript. CN reviewed and edited the manuscript. PW reviewed and edited the manuscript. PW conceived the idea. All authors contributed to the article and approved the submitted version.

## Funding

MA is supported by the National Institutes of Health (NIH) grant R01GM129633, and PW is supported by NIH grants R35GM118337, R01HL076179, R01AA028947 and U01AI133655.

## Conflict of Interest

The authors declare that the research was conducted in the absence of any commercial or financial relationships that could be construed as a potential conflict of interest.

## Publisher’s Note

All claims expressed in this article are solely those of the authors and do not necessarily represent those of their affiliated organizations, or those of the publisher, the editors and the reviewers. Any product that may be evaluated in this article, or claim that may be made by its manufacturer, is not guaranteed or endorsed by the publisher.

## Abbreviations

CLP, cecal ligation and puncture; DAMPs, damage-associated molecular patterns; eCIRP, extracellular cold-inducible RNA-binding protein; AKI, acute kidney injury; ALI, acute lung injury; ARDS, acute respiratory distress syndrome; GPX4, glutathione peroxidase 4; TLR4, Toll-like receptor 4; TREM-1, triggering receptor expressed on myeloid cells-1; NETs, neutrophil extracellular traps; HMGB1, high-mobility group box-1.
